# Case Report: Novel Disseminated *Paecilomyces formosus* Infection in a Dog

**DOI:** 10.3389/fvets.2022.878327

**Published:** 2022-05-17

**Authors:** Stephanie Anderson, Daniel Felipe Barrantes Murillo, Mandy Womble, Nicole Gibbs, Karyn Harrell, Tatiane Terumi Negrão Watanabe

**Affiliations:** ^1^Department of Population Health and Pathobiology, College of Veterinary Medicine, North Carolina State University, Raleigh, NC, United States; ^2^Department of Pathobiology, College of Veterinary Medicine, Auburn University, Auburn, AL, United States; ^3^Department of Clinical Sciences, College of Veterinary Medicine, North Carolina State University, Raleigh, NC, United States

**Keywords:** *Paecilomyces formosus*, hyalohyphomycosis, systemic mycosis, domestic animal, dog

## Abstract

A 2.5-year-old, 25.5 kg, spayed female Australian Shepherd dog had a 2-month history of shifting leg lameness in all limbs, tetraparesis, progressive lethargy, and severe pain. On the physical examination, fever (40.61°C), tachycardia, tachypnea, mild diffuse pelvic limb muscular atrophy, left prescapular and right popliteal lymphadenomegaly were observed. Due to the poor prognosis and difficult pain management, humane euthanasia was elected. Macroscopic and histological findings revealed multifocal to coalescing granulomas with central areas of lytic necrosis within the right femur, left humerus, left scapula, left biceps brachii, right semimembranosus muscle, liver, spleen, and lymph nodes. The necrotic areas contained myriad intralesional, intracellular, and extracellular negatively stained, non-pigmented, septate acute angle branching hyphae with parallel walls measuring 3–6 μm in width with polar bulbous projections measuring 7–13 μm in width. Fresh samples of the liver were submitted for fungal culture. Panfungal PCR targeting the major conserved genes-ITS, TUB, CAL-confirmed *Paecilomyces formosus. Paecilomyces* spp. are members of anamorphic fungi classified under the phylum Ascomycota. Paecilomycosis is an uncommon fungal infection caused by *Paecilomyces* spp with a disease reported in humans and animals ranging from superficial to systemic clinical forms affecting both immunocompromised and immunocompetent individuals. In dogs, disseminated paecilomycosis has been reported, but the species of fungi are not always determined. To our knowledge, this is the first case of disseminated paecilomycosis caused by *P. formosus* infection in a dog.

## Introduction

*Paecilomyces* spp. are members of anamorphic fungi classified under the phylum Ascomycota that are commonly found in the soil, air, and decaying food (1, 2). Being saprophytic fungal organisms, *Paecilomyces* spp. are associated with hyalohyphomycosis, an infection caused by non-pigmented filamentous molds (3). Other fungal agents associated with hyalohyphomycosis include *Aspergillus* spp., *Fusarium* spp., and *Pseudallescheria* spp. (4). Paecilomycosis is a rare fungal infection caused by *Paecilomyces* species with the disease ranging from a superficial to systemic clinical form affecting both immunocompromised and immunocompetent individuals (1, 5). However, in domestic animals, it is still unclear if the infection is solely an opportunistic disease. Approximately 15 species of *Paecilomyces* spp. have been reported acting as the causative agents of clinical disease in vertebrates (4) with *Paecilomyces variotii* and *Purpureocillium lilacinus* (formerly known as *Paecilomyces lilacinus)* being the most commonly reported in both humans and animals (4, 6, 7).

## Case Presentation

A 2.5-year-old, 25.5 kg, spayed female Australian Shepherd dog had a 2-month history of shifting leg lameness in all limbs, tetraparesis, progressive lethargy, and severe pain. During that time, the dog was previously treated with non-steroidal anti-inflammatory drugs (NSAIDs) deracoxib (2 mg/kg PO 24 h), meloxicam (0.1 mg/kg PO q 24 h), and doxycycline (10 mg/kg PO q 24 h) for polyarthritis of unknown etiology. After 10 days of treatment with NSAIDs, the patient was not responsive so a high immunosuppressive dose of prednisone (2.4 mg/day q 24 h) was applied for pain management, for 6 weeks. Because of the improved clinical signs and in order to avoid the side effects-especially polyuria and polydipsia-the prednisone dose was decreased until 0.4 mg/day q 24 h. However, the clinical signs worsened with severe pain on lower doses of prednisone. On the physical examination, fever (40.61°C), tachycardia, tachypnea, and mild diffuse pelvic limb muscular atrophy were observed. The left prescapular and right popliteal lymph nodes were markedly enlarged, and bilateral stifle effusion was observed; however, arthrocentesis was not performed. Severe cervical, thoracolumbar, and left scapular pain were noted. The dog was hospitalized and fluid therapy of 0.45% NaCl maintenance fluid rate + 0.05 mEq/kg/h KCl was administered. Fentanyl (up to 4 mcg/kg/h) and ketamine (up to 3 mcg/kg/min) were prescribed for analgesia. Spinal radiographs were taken and highlighted a polyostotic effacing lytic osseous lesion on the left distal scapula and multifocal lymphadenopathy of medial iliac lymph nodes. At the time of hospitalization a complete blood count (CBC) revealed mild normocytic, normochromic, regenerative anemia (MCV 69.1 fL, reference value 64.9–75.2 fL; MCH 23.2 pg, reference value 21.8–25.7 pg; HCT 23.5%, reference value 40.2–61%; HGB 7.9 g/dl, reference value 13.7–21g/dl, Reticulocytes 2.34%, reference value 0.11–1.26%, moderate thrombocytopenia (109 × 10^3^/UL; reference value 190–468 × 10^3^/UL), leukocytosis (21.63 × 10^3^/UL; reference value 4.36–11.9 × 10^3^/UL) with neutrophilia (17.73 × 10^3^/UL; reference value 2.84–9.11 × 10^3^/UL). Serum biochemistry profile showed mild hypoalbuminemia (2.9 g/dl; reference value 3.2–4.3 g/dl), mild hyperglobulinemia (3.5 g/dl; reference value 1.8–3.4 g/dl), and a cholestatic pattern characterized by elevated ALP (430 IU/L; reference value 9–88 IU/L), GGT (9 IU/L; reference value 0–4 IU/L) and AST (66 IU/L; reference value 9–88 IU/L). Due to the poor prognosis and difficult pain management, no further diagnostic testing was performed and humane euthanasia was elected.

**Figure 1:**
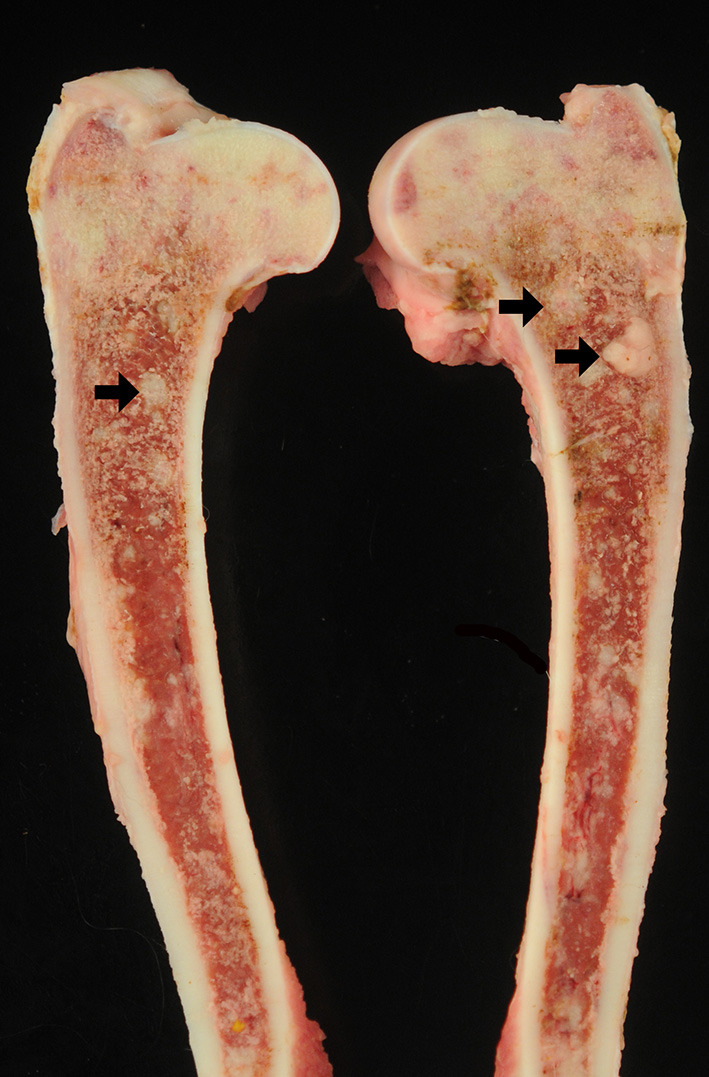
Photographs from gross postmortem examination of a 2.5-year-old, female spayed, Australian Shepherd dog that had a 2-month history of tetraparesis, progressive lethargy, and severe pain. The bone marrow and trabecular bone from both femurs were replaced and expanded by multifocal to coalescing pale tan, soft, nodules ranging from pinpoint to 2.0 cm in diameter (arrows).

Grossly, the head of both humeri and femurs (Figure 1), and the L7-S1 intervertebral joints were red, roughened, and fragmented with exposure of the subchondral bone. Pathological fractures with associated small amounts of green to gray, granular exudate was also noted. The articular surface of the left scapula was roughened with articular capsular fibrosis and dark red connective tissue overlying the supraglenoid tubercle. The femoral bone marrow was light red with coalescing pale tan nodules. The mediastinal, tracheobronchial, prescapular, sub-lumbar, and iliac lymph nodes were markedly enlarged ranging from 1.2 × 2.3 × 1.3 cm to 7.2 × 3 × 2.4 cm. On cut surface, there was loss of the corticomedullary junction, and the nodal architecture was replaced by irregularly shaped, mottled pale tan to light pink masses. The liver (6.5% body weight; reference value: 3–4%) and spleen were markedly enlarged with random miliary, pinpoint pale tan nodules that extended into the parenchyma. An enhanced reticular pattern was observed on the liver, and the spleen had a meaty consistency. Samples of multiple organs were fixed in 10% neutral buffered formalin, processed routinely, and stained with hematoxylin and eosin (H&E). Fresh samples of the liver and mediastinal lymph nodes were submitted for aerobic and fungal culture.

**Figure 2:**
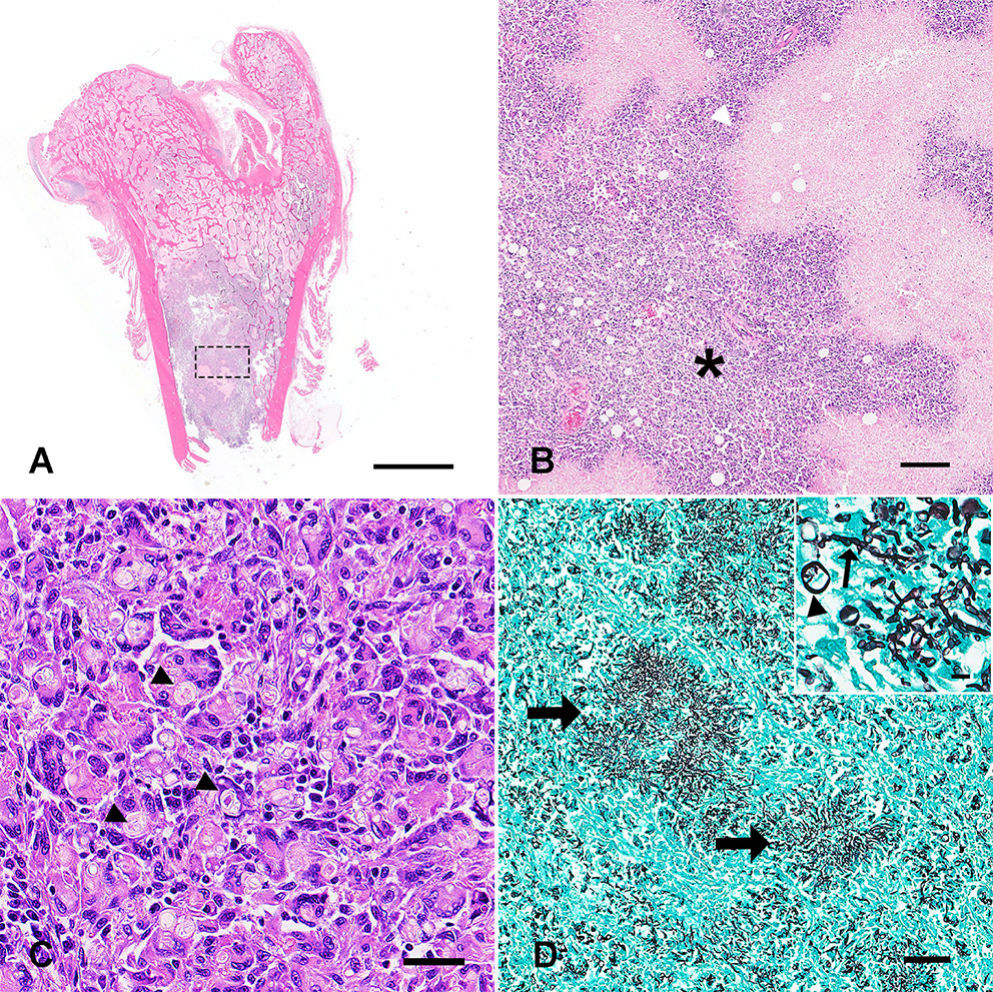
Photomicrographs of sections of the femur **(A,B)** and lymph node **(C,D)** obtained from the dog in Figure 1. **(A)** The marrow space was largely effaced and replaced by multifocal to coalescing granulomatous cellular infiltrate (H&E; bar = 9 mm). **(B)** Higher magnification of the granulomas from **A** (dashed square). The granulomas were centered on extensive areas of lytic necrosis (white arrowhead) and dissected by myriad macrophages and epithelioid macrophages, numerous multinucleated giant cells with up to 10 nuclei, and moderate numbers of lymphocytes, plasma cells, and lesser neutrophils (asterisk; H&E; bar = 200 μm). **(C)** The lymph node architecture was completely replaced by similar cellular infiltrates. Numerous intralesional, intracellular and extracellular, negatively stained, non-pigmented, septated with parallel walls, acute angle branching hyphae measuring 3–6 μm in width with polar bulbous projections measuring 7–13 μm in width (arrowheads; H&E; bar = 50 μm). **(D)** Hyphal mats were positively stained with Gomori methenamine-silver stain (arrows) (bar = 100 μm). Occasionally, yeast forms measuring 7–15 μm were present (arrowhead) among hyphae (arrow) (inset; bar = 20 μm).

Histologically, the normal architecture of the right femur, left humerus, left scapula, left biceps brachii, right semimembranosus muscle, liver, spleen, and lymph nodes were nearly completely effaced and replaced by multifocal to coalescing granulomas with central areas of lytic necrosis. The necrotic areas contained myriad intralesional, intracellular and extracellular, negatively stained, non-pigmented, septate acute angle branching hyphae with parallel walls measuring 3–6 μm in width with polar bulbous projections measuring 7–13 μm in width (Figures 2A–C). Occasionally, yeast-like forms measuring 7–15 μm were present. Hyphae positively stained with Gomori methenamine-silver (GMS) and Periodic acid-Schiff (PAS) stains (Figure 2D). The granulomas were surrounded by epithelioid macrophages, numerous multinucleated giant cells with up to 10 nuclei, and moderate numbers of lymphocytes, plasma cells, and fewer neutrophils (Figure 2C). The cellular infiltrates along the left biceps brachii and right semimembranosus muscles extended to the surrounding tissues causing secondary suppurative steatitis and myositis. Aerobic bacterial culture did not reveal any bacterial growth. A sample of fresh liver tissue was submitted for fungal culture and the isolated mold was submitted for panfungal PCR targeting the major conserved genes: internal transcribed spacer (ITS), β-tubuline (TUB), and calmodulin (CAL). The PCR product was purified from the gel, sequenced, and identified using the Basic Local Alignment Search Tool (BLAST) at the NCBI database of ITS sequences. The sequences matched with *Paecilomyces formosus* with 100% identitified for ITS, 100% for TUB, and 99% for CAL. PCR and sequencing in combination with histology aided a diagnosis of systemic *P*. *formosus* infection in a dog.

## Discussion

*P. formosus* infection in humans rarely occurs and has only been reported a few times in the literature (1, 8, 9), with the first report of *P. formosus* infection in human patients in 2015 (9). In immunocompromised human patients, infection by *Paecilomyces. variotii* and *Purpureocillium lilacinus* have been associated with pneumonia, dermatitis, ocular infections, peritonitis, and disseminated mycosis (4, 6). *P. formosus* has been described causing fibrosis of the lungs characterized by diffuse lobular consolidations and fine nodules in a patient with chronic granulomatous disease (CGD) (8), cutaneous infection with yellow-brown nodules in an extremely premature infant (9), and organizing pneumonia in an immunocompetent patient (1).

The transmission in immunocompetent humans has been reported secondary to invasive surgical procedures such as organ transplantation, prosthetic implantation, and dialysis (1). Whereas, inhalation, dermal exposure by a traumatic event, and lacrimal duct catheter placement are considered to be the routes of transmission in immunocompromised individuals (6). In dogs and cats, it is speculated that the soil provides a route of exposure since *Paecilomyces* spp is a common environmental organism (5). Skin wounds and the respiratory tract can also allow localized or systemic hyalohyphomycosis in the cat and the dog, similar to humans (4). *Paecilomyces* spp infections in veterinary patients are seldom reported. Comorbidities and immunocompromising factors reported in dogs include neoplasia (nasal carcinoma), experimental coinfections, steroid therapy, skin trauma, diarrhea, and otitis (4). However, the majority of reported cases are in immunocompetent animals, suggesting that most cases may be caused by a natural infection without an underlying immunosuppression factor.

Paecilomycosis is seldom reported in veterinary medicine literature and the clinical presentation varies from localized to systemic infection as similarly observed in humans (4). Across all animals, *Paecilomyces* spp infections are more frequently reported in dogs, and *P. lilacinus* and *P. variotii* are the two most common species observed (4, 10); however, to this date, *P*. *formosus* infection has not been identified in dogs. Clinically, the systemic disseminated form of the disease presents as: discospondylitis, with involvement of internal organs including spleen, liver, lymph nodes; pneumonia (4); lameness; and lethargy (10). Whereas, the localized form in dogs consists of discospondylitis, prostatitis, cystitis, and rhinitis without evidence of disseminated fungal infection (4). Other clinical presentations include invasive cutaneous and pulmonary infections in cats caused by *P. lilacinus* (5, 11), *P. viridis* causing disseminated mycosis in chameleons (*chamaeleo calyptratus*) (12), keratitis and pneumonia in horse species unidentified (4), and cutaneous mycosis in soft-shelled turtles (*Trionyx sinensis*) from *Purpureocillium lilacinus* (13). To our knowledge, this is the first report that describes a rare *P*. *formosus* infection causing systemic paecilomycosis in a dog. The immunocompetence of this animal at presentation, when clinical signs were first observed, was not determined; however, subsequent immunosuppressive doses of prednisone for 6 weeks likely played a crucial role in fungal dissemination in this particular case.

For this case, mold isolated from fungal culture was submitted for identification via pan-fungal PCR followed by DNA sequencing that identified *P. formosus*. Panfungal PCR targets the ITS2 region of rDNA in various fungal species and is able to amplify all fungal DNA present in a sample due to the broad range assay (14, 15). Molecular-based techniques have become more common in recent years due to their increased sensitivity and fast results ensuring better reliability when differentiating between fungal organisms. This is especially relevant in this case since *Paecilomyces* spp. can share a strong morphological resemblance to *Penicillium* spp. on culture (1). Definitive fungal identification is essential for appropriate antifungal treatment (1, 6).

Based on the progressive pain, lameness, lethargy, aggressive lytic lesions affecting multiple appendicular bones, and gross findings, differential diagnoses included neoplasia such as round cell tumors, systemic mycoses including aspergillosis, and a systemic bacterial infection most likely caused by *Escherichia coli* (16).

Due to the non-specific clinical presentation, a variety of broad range diagnostic tests such as radiographs, urinalysis, blood tests (CBC, biochemical, and serological), and fungal culture are often needed to reach an antemortem diagnosis of systemic paecilomycosis. Light microscopy and fungal culture are the common diagnostic tools used to identify *Paecilomyces* spp; however, some isolates from clinical samples grow atypically on inappropriate agars or become atypical when antimycotics are used. Thus, molecular-based diagnostic tools such as PCR and sequencing are more reliable and appropriate for determining specific fungal species (6). For the antemortem or postmortem identification of *Paecilomyces* spp., blood agar (BA), Sabouraud dextrose agar (sab-dex), and malt extract agar (MEA) (2) have been used with success (8). Creatine agar (CREA) plates specifically aid in the identification of *P. formosus* by distinguishing it from *P. variotii* (2). In mycological culture, the color and colony growth are highly variable between species (17). *Paecilomyces* spp. colonies are floccose and white; later, the color changes and texture is described as wooly to powdery in Sabouraud dextrose agar without cycloheximide (18). *P. variotti* colonies are velvety and tan olive-brown, whereas *P. lilacinus* is pink or vinaceous to lilac (18). Colonies of *P. formosus* will exhibit a yellowish-gray color on blood agar (10). Microscopically, *P. formosus* has single branching phialides resembling a brush, with long chains of 3–10 × 1.8–3.5 μm ellipsoidal to cylindrical conidia with truncate ends, with numerous chlamydospores present and without ascospores (1, 2, 8). Differentiation between *P. formosus* and *P. variotii* is not possible since both look similar on histological evaluation. In culture *P. formosus* will produce acid when grown on CREA and exhibits faster growth at milder temperatures (30°C); whereas *P. variotii* grows faster at warmer temperatures (37°C) and does not produce acid when grown on CREA (2, 6).

Microscopically, *Paecilomyces* spp. are described within the group of hyaline septate molds, and share similar morphological and clinical features along with *Aspergillus* spp., *Fusarium* spp., *Scedosporium* spp., *Trichoderma* spp., *Scopulariopsis* spp., *Acremonium* spp., *Schizophyllum* spp., *Phaeoacremonium* spp., and *Trichosporon* spp. (19). The histologic findings are similar to *Aspergillus* spp. and other hyaline septate mold infections, and the morphology described on HE, GMS, and PAS staining is characterized by the presence of non-pigmented (hyaline), septate hyphae with acute angle branching (19). In some instances, the hyphae of these organisms have a globose appearance, as in this case. When this feature is present, the *Mucorales* genera should be included as a differential diagnosis, and demonstration of septation is necessary (19). *Paecilomyces* spp. histological description in dogs includes branching fungal hyphae (10). One case of *Purpureocillium lilacinus* in a cat causing cutaneous infection described the presence of pseudohyphae and round yeast-like structures measuring 5 to 8 μm in diameter (5). In the case presented in this report, yeast-like forms were present along with the globose hyphae.

Dogs with disseminated paecilomycosis generally have a poor prognosis and either succumb to the disease or are euthanized (4), as reported herein. In domestic animals such as dogs and cats, due to the sporadic and progressive nature of the disease, early diagnosis is often challenging; therefore, early effective antifungal treatment is often not pursued (10). Itraconazole was used successfully to treat cutaneous paecilomycosis in a domestic cat infected with *Purpureocillium lilacinus* (5). In humans, treatment with amphotericin B (8) and micafungin (9) have been reported with successful results in cases of *P. formosus* infection. In all three cases of human patients infected with *P. formosus*, the antifungal treatment was eventually curative of the disease and the patient made a full recovery (1, 8, 9). It is speculated that there is a better prognosis in humans than in veterinary medicine mainly because of an early diagnosis and fungal prophylaxis. However, since cases of *Paecilomyces* spp. causing invasive fungal infections in domestic animals are infrequent, there is a lack of understanding of the pathogenesis and therapeutic protocols in veterinary medicine. The increase in the early diagnosis of systemic paecilomycosis in animals will contribute to refining the diagnostic and therapeutic approach.

## Conclusion

In dogs, disseminated paecilomycosis has been previously reported, but the species of fungi are not always determined. To our knowledge, this case is the first report of disseminated paecilomycosis caused by *P. formosus* infection in a dog associated with severe multifocal osteomyelitis, pathological fractures, and granulomatous systemic disease in multiple organs excluding the lungs. With the continued development in diagnostic techniques and the use of molecular-based methods, it is likely more cases with *Paecilomyces* spp. infection will be reported with species-specific identification. This case demonstrates that *P. formosus* infection is clinically significant and should be considered a differential diagnosis in veterinary patients with disseminated hyalohyphomycosis in a clinical setting.

## Data Availability Statement

The original contributions presented in the study are included in the article/supplementary material, further inquiries can be directed to the corresponding author/s.

## Author Contributions

NG and KH followed the clinical case. MW and TN performed the post-mortem examination, sample collection, and final post-mortem report. SA and DB prepared the manuscript and literature review. TN contributed to the design, supervised the study, and critically revised and edited the manuscript. SA, DB, MW, NG, KH, and TN reviewed the final submission. All authors read and approved the final manuscript.

## Conflict of Interest

The authors declare that the research was conducted in the absence of any commercial or financial relationships that could be construed as a potential conflict of interest.

## Publisher's Note

All claims expressed in this article are solely those of the authors and do not necessarily represent those of their affiliated organizations, or those of the publisher, the editors and the reviewers. Any product that may be evaluated in this article, or claim that may be made by its manufacturer, is not guaranteed or endorsed by the publisher.
